# Phylogenomic Study of *Burkholderia glathei*-like Organisms, Proposal of 13 Novel *Burkholderia* Species and Emended Descriptions of *Burkholderia sordidicola, Burkholderia zhejiangensis*, and *Burkholderia grimmiae*

**DOI:** 10.3389/fmicb.2016.00877

**Published:** 2016-06-08

**Authors:** Charlotte Peeters, Jan P. Meier-Kolthoff, Bart Verheyde, Evie De Brandt, Vaughn S. Cooper, Peter Vandamme

**Affiliations:** ^1^Laboratory of Microbiology, Department of Biochemistry and Microbiology, Ghent UniversityGhent, Belgium; ^2^Leibniz Institute DSMZ–German Collection of Microorganisms and Cell Cultures GmbHBraunschweig, Germany; ^3^Department of Microbiology and Molecular Genetics, University of Pittsburgh School of MedicinePittsburgh, PA, USA; ^4^BCCM/LMG Bacteria Collection, Department of Biochemistry and Microbiology, Ghent UniversityGhent, Belgium

**Keywords:** *Burkholderia*, genomic taxonomy, GBDP, GGDC, MLSA, phylogenomics

## Abstract

Partial *gyrB* gene sequence analysis of 17 isolates from human and environmental sources revealed 13 clusters of strains and identified them as *Burkholderia glathei* clade (BGC) bacteria. The taxonomic status of these clusters was examined by whole-genome sequence analysis, determination of the G+C content, whole-cell fatty acid analysis and biochemical characterization. The whole-genome sequence-based phylogeny was assessed using the Genome Blast Distance Phylogeny (GBDP) method and an extended multilocus sequence analysis (MLSA) approach. The results demonstrated that these 17 BGC isolates represented 13 novel *Burkholderia* species that could be distinguished by both genotypic and phenotypic characteristics. BGC strains exhibited a broad metabolic versatility and developed beneficial, symbiotic, and pathogenic interactions with different hosts. Our data also confirmed that there is no phylogenetic subdivision in the genus *Burkholderia* that distinguishes beneficial from pathogenic strains. We therefore propose to formally classify the 13 novel BGC *Burkholderia* species as *Burkholderia arvi* sp. nov. (type strain LMG 29317^T^ = CCUG 68412^T^), *Burkholderia hypogeia* sp. nov. (type strain LMG 29322^T^ = CCUG 68407^T^), *Burkholderia ptereochthonis* sp. nov. (type strain LMG 29326^T^ = CCUG 68403^T^), *Burkholderia glebae* sp. nov. (type strain LMG 29325^T^ = CCUG 68404^T^), *Burkholderia pedi* sp. nov. (type strain LMG 29323^T^ = CCUG 68406^T^), *Burkholderia arationis* sp. nov. (type strain LMG 29324^T^ = CCUG 68405^T^), *Burkholderia fortuita* sp. nov. (type strain LMG 29320^T^ = CCUG 68409^T^), *Burkholderia temeraria* sp. nov. (type strain LMG 29319^T^ = CCUG 68410^T^), *Burkholderia calidae* sp. nov. (type strain LMG 29321^T^ = CCUG 68408^T^), *Burkholderia concitans* sp. nov. (type strain LMG 29315^T^ = CCUG 68414^T^), *Burkholderia turbans* sp. nov. (type strain LMG 29316^T^ = CCUG 68413^T^), *Burkholderia catudaia* sp. nov. (type strain LMG 29318^T^ = CCUG 68411^T^) and *Burkholderia peredens* sp. nov. (type strain LMG 29314^T^ = CCUG 68415^T^). Furthermore, we present emended descriptions of the species *Burkholderia sordidicola, Burkholderia zhejiangensis* and *Burkholderia grimmiae*. The GenBank/EMBL/DDBJ accession numbers for the 16S rRNA and *gyrB* gene sequences determined in this study are LT158612-LT158624 and LT158625-LT158641, respectively.

## Introduction

The genus *Burkholderia* currently comprises 90 validly named species (Euzeby, 1997) and several uncultured *Candidatus* species (Van Oevelen et al., 2004; Verstraete et al., 2011; Lemaire et al., 2012) which occupy very diverse niches (Coenye and Vandamme, 2003). Many *Burkholderia* species have thus far only been isolated as free-living organisms but a growing body of literature reveals that they live in close interaction with numerous plant, animal, fungal or even amoebozoan hosts (Marolda et al., 1999; Van Borm et al., 2002; Kikuchi et al., 2011; Verstraete et al., 2013; Stopnisek et al., 2016; Xu et al., 2016). *Burkholderia* species may be beneficial to their hosts because some strains can fix nitrogen, produce plant hormones or siderophores, or lower pathogen-related ethylene levels; hence they have been exploited for plant growth promotion and biocontrol of plant diseases (Compant et al., 2008; Vial et al., 2011). Yet, other *Burkholderia* species are notorious pathogens in plants, animals and humans (Mahenthiralingam et al., 2008). This ecological diversity is likely attributed to their large, multireplicon genomes (typically between 6 and 9 Mb) which also confer a metabolic versatility allowing them to degrade a wide range of recalcitrant xenobiotics (Parke and Gurian-Sherman, 2001; Coenye and Vandamme, 2003).

Phylogenetic analyses based on the 16S rRNA and protein-coding genes showed that *Burkholderia glathei* clade (BGC) species are phylogenetically divergent from other *Burkholderia* species and form a separate clade (Sawana et al., 2014; Vandamme et al., 2014). Although this clade thus far includes only 12 formally named species, its functional diversity is impressive. In this clade too, most species have been isolated from bulk and rhizosphere soil (Zolg and Ottow, 1975; Viallard et al., 1998; Vandamme et al., 2013; Draghi et al., 2014; Baek et al., 2015), but also from contaminated soil and sludge from a wastewater treatment system (Lu et al., 2012; Vandamme et al., 2013; Liu et al., 2014). Two BGC species were associated with less studied hosts like fungi (*Burkholderia sordidicola*) and mosses (*Burkholderia grimmiae*) (Lim et al., 2003; Tian et al., 2013) but numerous, mostly uncultivated BGC species adopted endosymbiotic lifestyles in insect guts (Kikuchi et al., 2011; Tago et al., 2015; Xu et al., 2016) or plant leaf tissue (Verstraete et al., 2013; Carlier et al., 2015) and many additional unclassified *B. glathei*-like bacteria have been reported (Nogales et al., 2001; Salles et al., 2006; Pumphrey and Madsen, 2008; Draghi et al., 2014; Verstraete et al., 2014; Peeters et al., 2016).

The present study aimed to perform a phylogenomic study of established and novel species in the *B. glathei* clade, to formally name the latter and to make reference cultures and whole-genome sequences of each of these versatile bacteria publicly available. The genome sequence-based phylogeny was assessed using the Genome Blast Distance Phylogeny (GBDP) method (Meier-Kolthoff et al., 2013) and an extended multilocus sequence analysis (MLSA) approach. For phenotypic characterization, whole-cell fatty acid profiling and biochemical analyses were performed.

## Materials and methods

### Bacterial strains and growth conditions

Table 1 lists the sources of the 17 studied isolates. Details of type strains of each of the present BGC species were described previously (Zolg and Ottow, 1975; Lim et al., 2003; Lu et al., 2012; Tian et al., 2013; Vandamme et al., 2013; Draghi et al., 2014; Liu et al., 2014; Baek et al., 2015). Strains were grown aerobically on buffered nutrient agar (Oxoid, pH 6.8) and incubated at 28°C. Cultures were preserved in MicroBank^TM^ vials at −80°C.

**Table 1:** **Strains included in the present study**.

**Strain**	**Other strains designations**	**Source**	**Depositor**	**References**
***Burkholderia arvi*** **sp. nov**.				
LMG 29317^T^	CCUG 68412^T^, MAN34^T^	Soil (Argentina, 2010)	Walter Draghi	Draghi et al., 2014
***Burkholderia hypogeia*** **sp. nov**.				
LMG 29322^T^	CCUG 68407^T^	Soil (Belgium, 2014)	Own isolate	Peeters et al., 2016
***Burkholderia ptereochthonis*** **sp. nov**.				
LMG 29326^T^	CCUG 68403^T^	Soil (Belgium, 2014)	Own isolate	Peeters et al., 2016
***Burkholderia glebae*** **sp. nov**.				
LMG 29325^T^	CCUG 68404^T^	Soil (Belgium, 2014)	Own isolate	Peeters et al., 2016
LMG 22938	RA57-7	Soil (Netherlands)	Joana Salles	Salles et al., 2006
***Burkholderia pedi*** **sp. nov**.				
LMG 29323^T^	CCUG 68406^T^	Soil (Belgium, 2014)	Own isolate	Peeters et al., 2016
R-52605		Soil (Belgium, 2014)	Own isolate	Peeters et al., 2016
***Burkholderia arationis*** **sp. nov**.				
LMG 29324^T^	CCUG 68405^T^	Soil (Belgium, 2014)	Own isolate	Peeters et al., 2016
R-23361	RG47-6	Soil (Netherlands)	Joana Salles	Salles et al., 2006
***Burkholderia fortuita*** **sp. nov**.				
LMG 29320^T^	CCUG 68409^T^	Soil (South Africa, 2013)	Brecht Verstraete	Verstraete et al., 2014
***Burkholderia temeraria*** **sp. nov**.				
LMG 29319^T^	CCUG 68410^T^	Soil (South Africa, 2013)	Brecht Verstraete	Verstraete et al., 2014
***Burkholderia calidae*** **sp. nov**.				
LMG 29321^T^	CCUG 68408^T^	Water (Belgium, 2013)	Own isolate	Peeters et al., 2016
***Burkholderia concitans*** **sp. nov**.				
LMG 29315^T^	CCUG 68414^T^, AU12121^T^	Lung tissue (USA, 2006)	John J. LiPuma	
R-46586	AU21394	Blood (USA, 2010)	John J. LiPuma	
***Burkholderia turbans*** **sp. nov**.				
LMG 29316^T^	CCUG 68413^T^, HI4065^T^	Pleural fluid (USA, 2006)	John J. LiPuma	
***Burkholderia catudaia*** **sp. nov**.				
LMG 29318^T^	CCUG 68411^T^	Soil (South Africa, 2013)	Brecht Verstraete	Verstraete et al., 2014
***Burkholderia peredens*** **sp. nov**.				
LMG 29314^T^	CCUG 68415^T^, NF100^T^	Soil (Japan)	M. Hayatsu	Hayatsu et al., 2000

*LMG, BCCM/LMG Bacteria Collection, Laboratory of Microbiology, Ghent University, Ghent, Belgium*.

### 16S rRNA gene sequence analysis

Nearly complete sequences were obtained as described previously (Peeters et al., 2013).

### *gyrB* gene sequence analysis

Partial *gyrB* gene sequences were obtained as described previously (Spilker et al., 2009; Peeters et al., 2013). Sequence assembly was performed using BioNumerics v7.5 (Applied Maths). Sequences (589–1182 bp) were aligned based on amino acid sequences using Muscle (Edgar, 2004) in MEGA6 (Tamura et al., 2013). All positions with less than 95% site coverage were eliminated, resulting in a total of 570 positions in the final dataset. Phylogenetic analysis was conducted in MEGA6 (Tamura et al., 2013).

### Whole-genome sequencing

Genomic DNA of 20 strains (Table 2) was prepared as described by Pitcher et al. (1989). Genomic libraries were prepared using the Nextera kit following the methods introduced by Baym et al. (2015) and the 151 bp paired-end libraries were sequenced on the Illumina HiSeq platform of the University of New Hampshire Hubbard Center for Genomics Studies with an average insert size of 386 bp. Quality reports were created by FastQC. Adaptors and low-quality reads were trimmed using Trimmomatic (Bolger et al., 2014) with the following options: ILLUMINACLIP:NexteraPE-PE.fa:2:30:10 MAXINFO:60:0.4 MINLEN:60. Assembly was performed using SPAdes (Bankevich et al., 2012) with default k-mer sizes (21, 33, 55, 77) and mismatch correction (option—careful). Contigs with length <500 bp and coverage <2 were discarded from the resulting assemblies. Raw reads were mapped against the assemblies using bwa-mem (Li, 2013) and contigs were polished using Pilon (Walker et al., 2014) with default parameters. Quast (Gurevich et al., 2013) was used to create quality reports of the resulting assemblies. Annotation was performed using Prokka 1.11 (Seemann, 2014) with a genus-specific database based on reference genomes from the *Burkholderia* Genome Database (Winsor et al., 2008).

**Table 2:** **Genomes included in the present study**.

**Strain**	**Project**	**Contigs^a^**	**Size (bp)**	**%GC**	**References**
*B. glathei* LMG 14190^T^	PRJEB6934	139	8,049,485	64.7	Stopnisek et al., 2016
*B. sordidicola* LMG 22029^T^	PRJEB12475	72	6,874,511	60.2	This study
*B. zhejiangensis* OP-1^T^	PRJNA238427	116	7,767,215	62.7	Liu et al., 2014
*B. grimmiae* R27^T^	PRJNA238424	160	6,704,301	63.0	Liu et al., 2014
*B. choica* LMG 22940^T^	PRJEB12479	657	9,776,207	62.7	This study
*B. humi* LMG 22934^T^	PRJEB12476	272	7,619,203	62.8	This study
*B. telluris* LMG 22936^T^	PRJEB12477	163	7,056,109	64.0	This study
*B. terrestris* LMG 22937^T^	PRJEB12478	645	8,201,357	62.6	This study
*B. udeis* LMG 27134^T^	PRJEB12480	242	10,051,569	60.0	This study
*B. cordobensis* LMG 27620^T^	PRJEB12481	74	8,208,096	63.7	This study
*B. jiangsuensis* MP-1^T^	PRJNA238425	168	8,611,053	62.6	Liu et al., 2014
*B. megalochromosomata* JC2949^T^	PRJNA241423^b^	285	9,506,519	62.7	Baek et al., 2015
*B. arvi* sp. nov. LMG 29317^T^	PRJEB12485	351	9,665,767	62.4	This study
*B. hypogeia* sp. nov. LMG 29322^T^	PRJEB12491	94	8,333,271	63.2	This study
*B. ptereochthonis* sp. nov. LMG 29326^T^	PRJEB12495	117	7,714,803	64.2	This study
*B. glebae* sp. nov. LMG 29325^T^	PRJEB12494	194	7,842,312	62.7	This study
*B. pedi* sp. nov. LMG 29323^T^	PRJEB12492	142	9,141,307	63.0	This study
*B arationis* sp. nov. LMG 29324^T^	PRJEB12493	629	9,377,494	62.8	This study
*B. fortuita* sp. nov. LMG 29320^T^	PRJEB12489	50	7,360,810	62.9	This study
*B. temeraria* sp. nov. LMG 29319^T^	PRJEB12488	129	8,325,519	62.7	This study
*B. calidae* sp. nov. LMG 29321^T^	PRJEB12490	379	9,609,693	62.5	This study
*B. concitans* sp. nov. LMG 29315^T^	PRJEB12483	47	6,166,171	63.2	This study
*B. turbans* sp. nov. LMG 29316^T^	PRJEB12484	120	7,352,555	63.1	This study
*B. catudaia* sp. nov. LMG 29318^T^	PRJEB12486	156	7,726,733	62.8	This study
*B. peredens* sp. nov. LMG 29314^T^	PRJEB12482	78	6,726,081	63.1	This study
*B. cordobensis* YI23	PRJNA74517	6	8,896,411	63.3	Lim et al., 2012
*Burkholderia* sp. PML1(12)	PRJNA53985	377	9,368,249	60.1	Uroz and Oger, 2015
*Burkholderia* sp. S170	PRJNA248610	216	10,261,891	59.6	Llado et al., 2014
*B. zhejiangensis* CEIB S4-3	PRJNA264584	154	7,666,841	62.8	Hernandez-Mendoza et al., 2014
*B. zhejiangensis* SJ98	PRJNA81431	14	7,878,727	62.7	Kumar et al., 2012
*Burkholderia* sp. Leaf177	PRJNA297956	27	6,804,288	59.2	Bai et al., 2015
*B. concitans* sp. nov. MR1	PRJNA269162	58	6,019,671	63.3	Pawitwar et al., 2015
*Burkholderia* sp. RPE64	PRJDB1103	5	6,964,487	63.2	Shibata et al., 2013
*B. cordobensis* RPE67	PRJDB1660	6	8,685,756	63.4	Takeshita et al., 2014
*Ca*. B. kirkii UZHbot1	PRJNA69825	305	3,990,738	62.9	Carlier and Eberl, 2012
*Ca*. B. kirkii UZHbot2	PRJNA253356	48	3,914,712	64.0	Pinto-Carbo et al., 2016
*Ca*. B. pumila UZHbot3	PRJNA253357	519	3,681,223	59.3	Pinto-Carbo et al., 2016
*Ca*. B. verschuerenii UZHbot4	PRJNA253359	446	6,188,480	61.9	Pinto-Carbo et al., 2016
*Ca*. B. humilis UZHbot5	PRJNA253360	354	5,148,994	60.1	Pinto-Carbo et al., 2016
*Ca*. B. calva UZHbot6	PRJNA253361	307	4,208,605	61.4	Pinto-Carbo et al., 2016
*Ca*. B. brachyanthoides UZHbot7	PRJNA253362	684	3,545,532	61.2	Pinto-Carbo et al., 2016
*Ca*. B. schumannianae UZHbot8	PRJNA253363	283	2,362,726	63.1	Pinto-Carbo et al., 2016
*Ca*. B. crenata UZHbot9	PRJNA253365	643	2,843,741	59.0	Carlier et al., 2015

a *Status complete: RPE64, RPE67, YI23; status draft assembly: all other genomes*.

b *Genome sequence not publicly available, contig sequences were provided by J. Chun (Baek et al., 2015)*.

### Publicly available genomes

Twenty three publicly available whole-genome sequences of BGC bacteria were downloaded from the NCBI database (Table 2). *B. gladioli* BSR3 (Seo et al., 2011) was used as an outgroup in all phylogenomic analyses. For *B. megalochromosomata* JC2949^T^ the whole-genome sequence was not publicly available (February 1st, 2016) and the contig sequences were provided by J. Chun (Baek et al., 2015). For *B. sordidicola* S170, *B. zhejiangensis* CEIB S4-3 and *B. megalochromosomata* JC2949^T^ no annotation was available and annotation was performed using Prokka as described above.

### Phylogenomic analysis

The latest version of the Genome Blast Distance Phylogeny (GBDP) approach was applied (Meier-Kolthoff et al., 2013) to calculate the intergenomic distance between each pair of genomes (based on the nucleotide data) and included the calculation of 100 replicate distances to assess pseudo-bootstrap support (Meier-Kolthoff et al., 2014a). Distance calculations were conducted under the recommended settings of the Genome-to-Genome Distance Calculator (GGDC 2.1; http://ggdc.dsmz.de), as described earlier (Meier-Kolthoff et al., 2013). The GBDP trimming algorithm and formula d_5_ were chosen because of their advantages for phylogenetic inference (Meier-Kolthoff et al., 2014a) and according distance matrices were prepared (a single matrix for the original distances plus 100 matrices containing the replicates). A phylogenomic tree with branch support (Meier-Kolthoff et al., 2014a) was inferred using FastME v2.07 with tree bisection and reconnection post-processing (Lefort et al., 2015). Moreover, pairwise digital DNA-DNA hybridization (dDDH) values and their confidence intervals were also determined using GGDC 2.1 under recommended settings (Meier-Kolthoff et al., 2013). The potential affiliation of the novel strains to existing species was determined by clustering using a 70% dDDH radius around each of the 12 BGC type strains as previously applied (Liu et al., 2015). Visualization and annotation of the phylogenetic tree was performed using iTOL (Letunic and Bork, 2011).

As an alternative for the GBDP method, an extended MLSA analysis was performed in which a whole-genome phylogeny was calculated based on single-copy orthologous genes as described previously (Pinto-Carbo et al., 2016). In short, single-copy orthologs were identified using blastp and OrthoMCL v2.0.9 (with *e*-value cutoff 1e10^−6^ and 50% match cutoff; Fischer et al., 2011) and aligned based on their amino acid sequences using MUSCLE. The alignments were trimmed using TrimAl (removing positions with gaps in more than 50% of the sequences) and concatenated to construct a Maximum Likelihood tree using RaXML v7.4.2 (Stamatakis, 2014) with the WAG amino acid substitution model and 100 rapid bootstrap analyses.

### Phenotypic characterization

Phenotypic and cellular fatty acid analyses were performed as described previously (Draghi et al., 2014).

## Results

### 16S rRNA gene sequence analysis

The 16S rRNA gene sequences determined in the present study are publicly available through the GenBank/EMBL/DDBJ accession numbers LT158612-LT158624.

### *gyrB* gene sequence analysis

**Figure 1:**
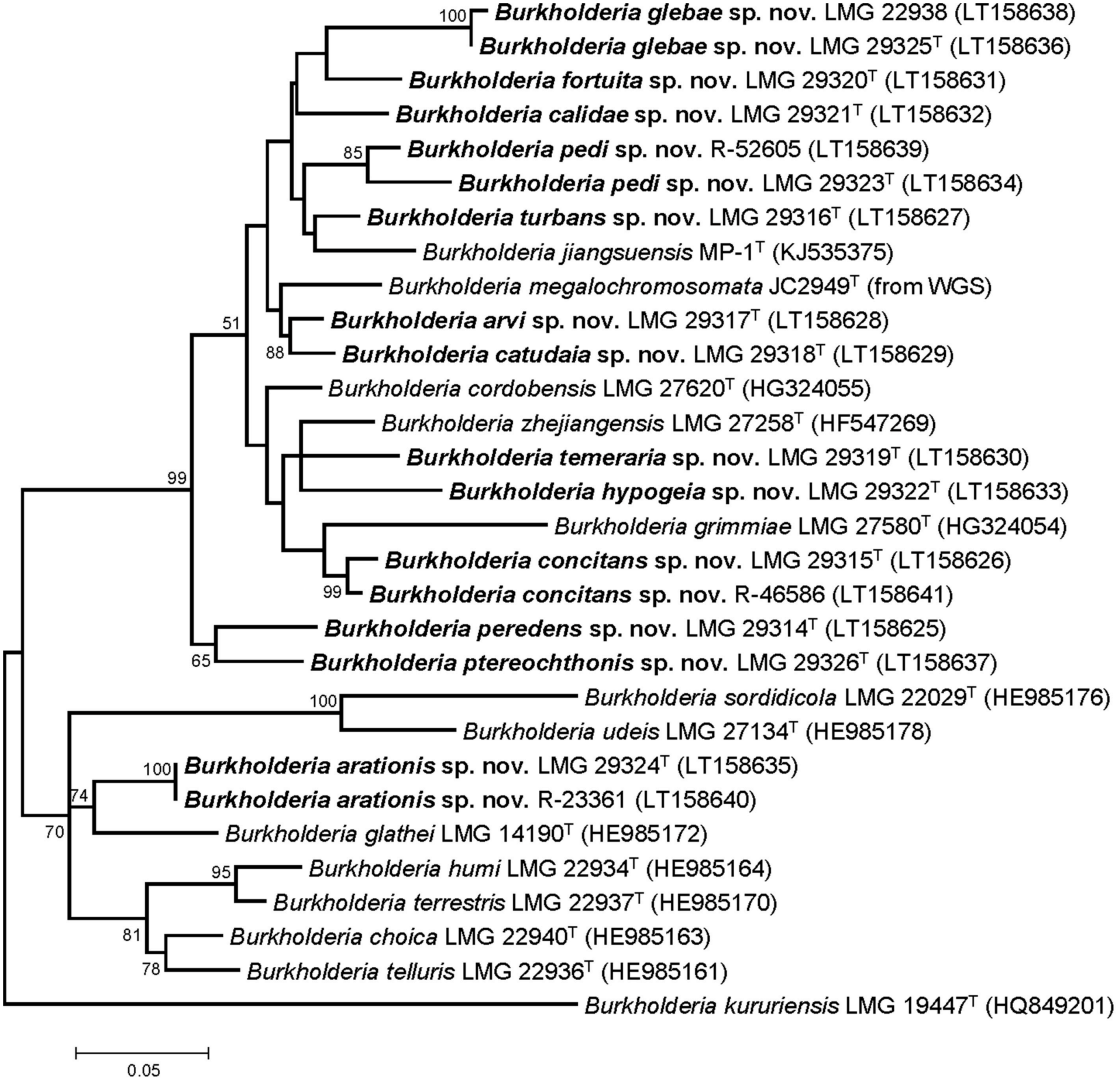
**Phylogenetic tree based on partial ***gyrB*** sequences of the 17 isolates in this study and type strains of phylogenetically related ***Burkholderia*** species**. The optimal tree (highest log likelihood) was constructed using the Maximum Likelihood method and General Time Reversible model in MEGA6 (Tamura et al., 2013). A discrete Gamma distribution was used to model evolutionary rate differences among sites [5 categories (+G, parameter = 0.5462)] and allowed for some sites to be evolutionarily invariable ([+I], 37.9331% sites). The percentage of replicate trees in which the associated taxa clustered together in the bootstrap test (1000 replicates) are shown next to the branches if greater than 50%. For *B. megalochromosomata* JC2949^T^ the *gyrB* gene sequence was extracted from the genome sequence. The *gyrB* sequence of *B. kururiensis* LMG 19447^T^ was used as outgroup. The scale bar indicates the number of substitutions per site.

Partial *gyrB* gene sequences were compared to those of the type strains of the 12 validly named BGC species (Figure 1). The 17 unclassified isolates represented 13 taxa which showed 83.4–96.2% pairwise identity with the *gyrB* sequences of the type strains of other BGC species. The *gyrB* gene sequences determined in the present study are publicly available through the GenBank/EMBL/DDBJ accession numbers LT158625-LT158641.

### Whole-genome sequencing

To further characterize the taxonomic status of these 13 taxa, we determined the whole-genome sequence of one strain per *gyrB* cluster and of *B. sordidicola* LMG 22029^T^, *B. choica* LMG 22940^T^, *B. humi* LMG 22934^T^, *B. telluris* LMG 22936^T^, *B. terrestris* LMG 22937^T^, *B. udeis* LMG 27134^T^, and *B. cordobensis* LMG 27620^T^. The assembly of the Illumina HiSeq 150 bp paired end reads resulted in assemblies with 47–657 contigs and a total of 6,166,171–10,051,569 bp (Table 2). The annotated assemblies of these 20 genomes were submitted to the European Nucleotide Archive and are publicly available through the GenBank/EMBL/DDBJ accession numbers listed in Table 2 and the species descriptions. The genome sequences of the remaining five BGC type strains and of 18 additional strains were publicly available (Table 2).

### DNA base composition

The G+C content of all type strains was calculated from their genome sequences and ranged from 62.4 to 64.2 mol% (Table 2).

### Phylogenomic analysis

**Figure 2:**
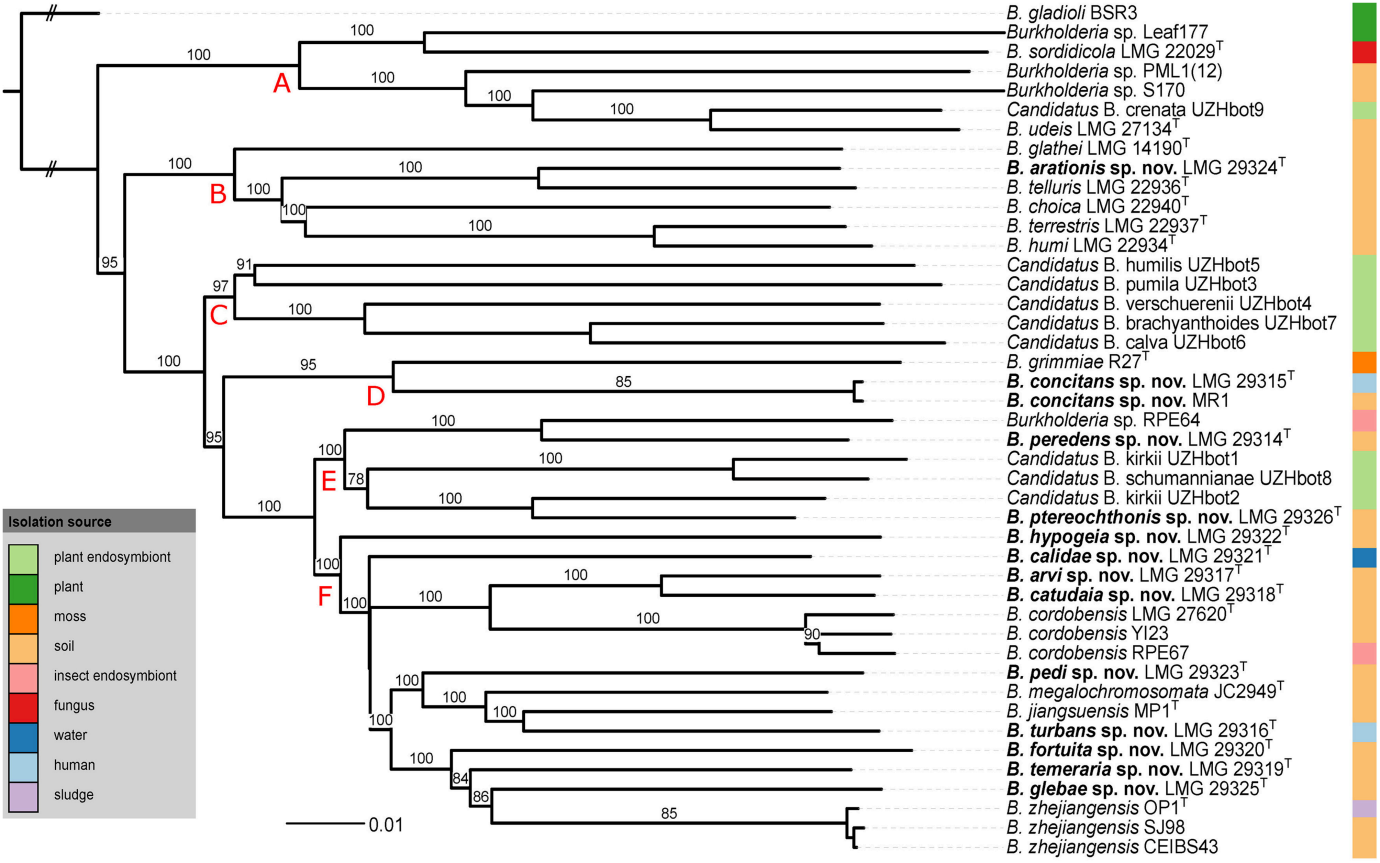
**Whole-genome sequence based phylogenomic tree of all BGC genomes inferred by GBDP**. The outer column shows the isolation source of the strains. Pseudo-bootstrap support values above 60% are shown. The tree reveals a high average support of 94.8%. Long terminal branches are due to the distinct scaling used by GBDP's formula d_5_. *B. gladioli* BSR3 was used as outgroup. Red capital letters define subtrees that also occur in the tree depicted in Figure 3.

The pairwise intergenomic distances and dDDH estimates of the 44 genome sequences are listed in Supplementary Table 1. The phylogenetic tree inferred from the intergenomic distances (Figure 2) was well resolved and most branches showed a very high bootstrap support (average support: 94.8%). Species delineation based on the pairwise dDDH values and a 70% dDDH radius around each type strain yielded 39 species which included the present 12 validly named species as well as the 13 novel species delineated by means of partial *gyrB* gene sequences (Figure 1).

**Figure 3:**
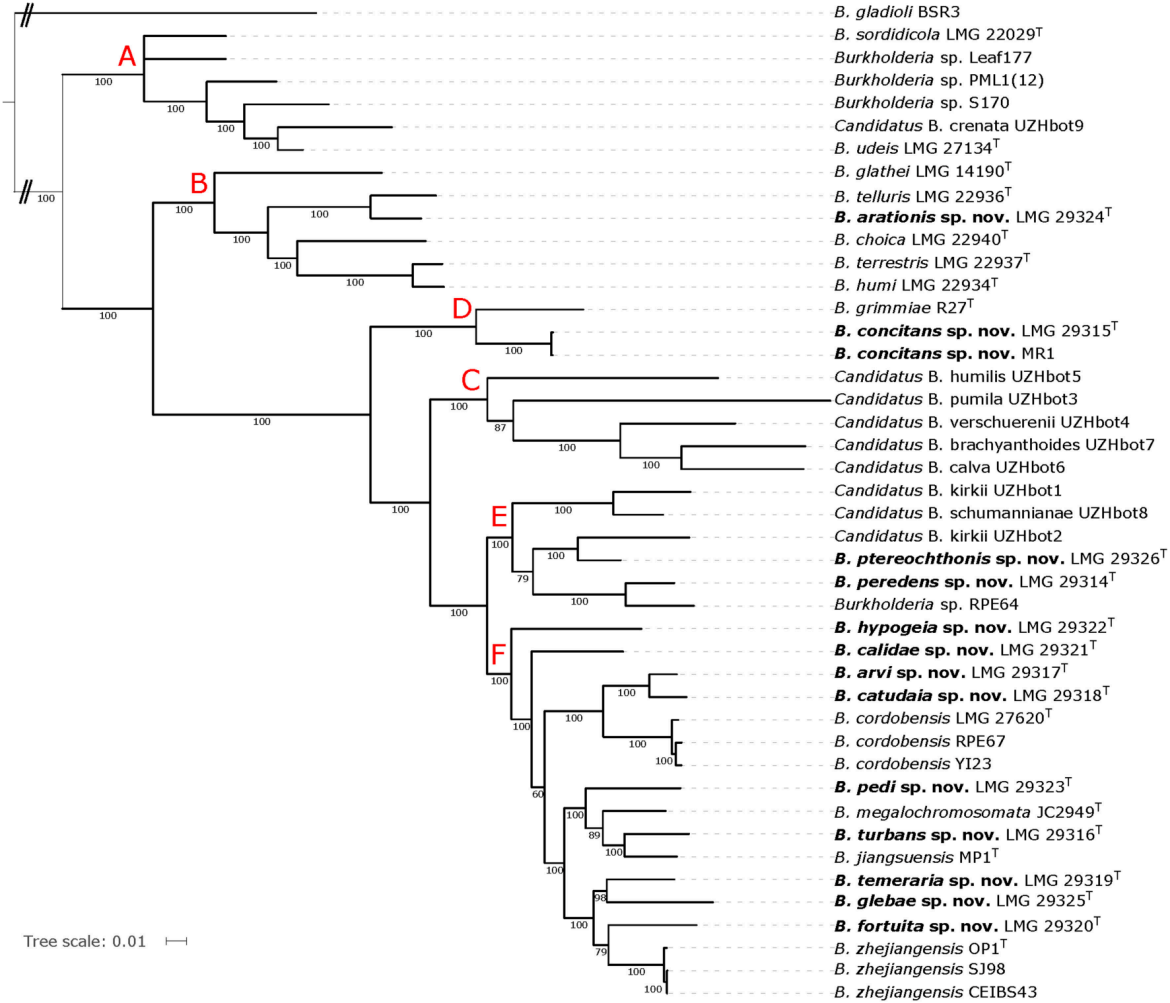
**Whole-genome phylogeny based on single-copy orthologs of all BGC genomes**. The phylogenetic tree was constructed using the WAG protein substitution model and RAxML and is based on an amino acid alignment with 105,141 positions from 332 single-copy orthologous genes. The percentage of replicate trees in which the associated taxa clustered together in the bootstrap test (100 replicates) are shown next to the branches if greater than 60%. *B. gladioli* BSR3 was used as outgroup. Red capital letters define subtrees that also occur in the tree depicted in Figure 2.

For the extended MLSA approach, we identified 332 single-copy orthologs that were present in all 44 genomes. The Maximum-Likelihood phylogenetic tree based on the concatenated amino acid alignment (Figure 3) was well resolved and showed a high bootstrap support on almost all branches.

The topologies of the two phylogenomic trees (Figures 2, 3) were very similar and both revealed six clusters of species (A-F). The main difference in tree topology related to the phylogenetic position of the *Candidatus* species in cluster C. This cluster was supported by a 100% bootstrap value in both analyses but its relative position to cluster D species differed in the two trees (Figures 2, 3). Additionally, the internal branching order of cluster C, E and F species differed minimally between both analyses. Both phylogenomic analyses showed that strain MR1 clustered with *B. concitans* sp. nov. and that strain RPE67 clustered with *B. cordobensis*. Finally, the large distances between strains PML1(12) and S170, and the type strains of *B. glathei* and *B. sordidicola*, respectively, indicated that both strains were misidentified and wrongly annotated in the NCBI database as *B. glathei* and *B. sordidicola*, respectively (Figures 2, 3). Both strains occupy unique positions in the phylogenomic trees and represent additional novel BGC species.

### Cellular fatty acid analysis

The fatty acid profiles of all strains are shown in Table 3. Branched chain fatty acids have not been reported in members of the genus *Burkholderia* and therefore summed features 2 and 3 very likely represent C_14:0_ 3-OH and C_16:1_ ω7c, respectively (Yabuuchi et al., 1992). The main fatty acid components are C_16:0_, C_18:1_ ω7c and summed feature 3 (most probably representing C_16:1_ ω7c).

Table 3**Mean fatty acid composition of all examined strains of BGC species**.

**Table:** 

**Fatty acid**	**1**	**2**	**3**	**4**	**5**	**6**	**7**	**8**	**9**	**10**	**11**	**12**
C_12:0_	ND	3.70	ND	TR	ND	ND	ND	ND	4.63 ± 0.07	ND	ND	1.01
C_14:0_	4.29 ± 0.13	0.79	4.08 ± 0.13	4.02	3.54 ± 0.13	3.71 ± 0.27	3.00 ± 0.01	3.80	TR	4.16 ± 0.04	4.36	3.97
C_16:0_	19.11 ± 1.93	18.29	16.24 ± 0.64	12.14	20.37 ± 4.40	12.00 ± 0.76	13.31 ± 0.15	16.02	15.72 ± 0.96	17.00 ± 0.80	15.58	15.23
C_16:0_ 2-OH	2.06 ± 0.86	3.32	3.37 ± 0.82	4.41	3.26 ± 1.54	2.43 ± 0.55	1.61 ± 0.14	2.29	2.05 ± 0.10	2.49 ± 0.79	4.10	1.53
C_16:0_ 3-OH	6.13 ± 0.49	4.90	5.91 ± 0.13	5.02	6.93 ± 2.32	5.17 ± 0.38	5.05 ± 0.04	5.36	6.75 ± 0.91	5.92 ± 0.04	6.44	4.89
C_16:1_ 2-OH	TR	4.06	TR	4.37	ND	1.71 ± 0.32	TR	1.29	2.02 ± 0.22	TR	1.77	TR
C_17:0_ cyclo	13.05 ± 2.41	13.94	4.96 ± 0.65	TR	14.30 ± 4.67	7.64 ± 2.05	4.54 ± 0.13	6.58	6.57 ± 0.99	5.31 ± 1.86	3.96	TR
C_18:1_ω7c	31.10 ± 1.83	27.61	36.88 ± 1.05	38.78	30.97 ± 1.41	42.19 ± 1.77	43.66 ± 0.55	38.00	32.56 ± 1.27	36.62 ± 1.20	35.48	42.25
C_19:0_ cyclo ω8c	2.64 ± 1.29	7.01	2.14 ± 0.37	ND	ND	TR	TR	ND	3.61 ± 0.68	1.82 ± 0.72	1.73	TR
Summed feature 2	7.08 ± 0.53	4.86	6.82 ± 0.35	5.32	7.80 ± 2.28	6.24 ± 0.42	5.71 ± 0.08	6.18	7.53 ± 0.60	7.21 ± 0.15	8.13	7.04
Summed feature 3	12.79 ± 2.79	11.53	17.43 ± 0.60	23.16	12.84 ± 4.21	17.14 ± 1.55	20.55 ± 0.57	18.98	17.20 ± 1.46	17.55 ± 2.11	18.44	20.64

**Table:** 

**Fatty acid**	**13**	**14**	**15**	**16**	**17**	**18**	**19**	**20**	**21**	**22**	**23**	**24**	**25**
C_12:0_	ND	ND	ND	TR	TR	ND	ND	ND	ND	TR	ND	ND	ND
C_14:0_	4.13	4.40	4.18	3.91 ± 0.13	4.11 ± 0.28	4.08 ± 0.78	4.36	4.21	2.15	4.40 ± 0.05	4.86	4.43	4.31
C_16:0_	15.87	18.25	16.71	15.38 ± 2.61	14.88 ± 0.02	17.09 ± 0.73	14.58	14.51	17.84	16.34 ± 1.82	26.27	16.97	15.11
C_16:0_ 2-OH	1.45	2.35	4.08	4.33 ± 0.53	3.31 ± 1.51	1.71 ± 1.71	3.45	3.68	2.48	7.31 ± 1.43	4.20	2.26	2.46
C_16:0_ 3-OH	5.28	5.64	6.94	5.63 ± 0.21	6.49 ± 0.16	10.29 ± 2.71	5.83	5.67	4.89	6.27 ± 1.50	4.68	5.16	6.27
C_16:1_ 2-OH	ND	TR	1.22	1.91 ± 0.05	1.27 ± 0.39	TR	1.22	TR	ND	1.99 ± 0.31	2.21	TR	1.52
C_17:0_ cyclo	4.01	6.77	9.10	5.97 ± 3.01	7.56 ± 4.61	7.65 ± 7.65	5.06	7.85	4.56	13.95 ± 3.77	13.76	3.64	2.02
C_18:1_ω7c	41.10	31.35	27.67	32.48 ± 4.27	32.00 ± 3.63	32.23 ± 4.96	34.89	35.05	41.75	23.16 ± 4.03	20.42	34.53	35.09
C_19:0_ cyclo ω8c	1.73	2.48	3.21	3.80 ± 1.45	1.96 ± 1.22	ND	1.64	4.40	TR	8.65 ± 4.56	3.60	1.20	ND
Summed feature 2	6.16	8.14	8.81	7.45 ± 0.86	8.73 ± 0.59	10.12 ± 2.55	7.13	6.84	6.12	6.45 ± 1.41	5.83	7.90	8.18
Summed feature 3	19.32	19.45	15.97	18.32 ± 0.18	18.44 ± 5.56	15.70 ± 5.44	20.31	15.52	18.83	8.82 ± 4.11	12.69	23.12	25.05

*Species: 1, B. glathei (5 strains); 2, B. sordidicola (1); 3, B. zhejiangensis (3); 4, B. grimmiae (1); 5, B. choica (2); 6, B. humi (6); 7, B. telluris (2); 8, B. terrestris (1); 9, B. udeis (3); 10, B. cordobensis (2); 11, B. jiangsuensis (1); 12, B. megalochromosomata (1); 13, Burkholderia arvi sp. nov. (1); 14, Burkholderia hypogeia sp. nov. (1); 15, Burkholderia ptereochthonis sp. nov. (1); 16, Burkholderia glebae sp. nov. (2); 17, Burkholderia pedi sp. nov. (2); 18, Burkholderia arationis sp. nov. (2); 19, Burkholderia fortuita sp. nov. (1); 20, Burkholderia temeraria sp. nov. (1); 21, Burkholderia calidae sp. nov. (1); 22, Burkholderia concitans sp. nov. (2); 23, Burkholderia turbans sp. nov. (1); 24, Burkholderia catudaia sp. nov. (1); 25, Burkholderia peredens sp. nov. (1). Data for B. glathei, B. sordidicola, B. zhejiangensis, B. choica, B. humi, B. telluris, B. terrestris and B. udeis were extracted from Vandamme et al. (2013). Data for B. cordobensis and B. grimmiae were extracted from Draghi et al. (2014). All other data are from the present study. Values are mean ± SD percentages of total fatty acids. Those fatty acids for which the mean amount for all taxa was <1% are not included, therefore, the percentages may not add up to 100%. TR, trace amount (<1 %); ND, not detected. Summed feature 2 comprises iso-C_16:1_I and/or C_14:0_ 3-OH; summed feature 3 comprises iso-C_15:0_ 2-OH and/or C_16:1_ω7c*.

### Biochemical characterization

An overview of biochemical characteristics useful for distinguishing the BGC species is shown in Table 4.

**Table 4:** **Differential biochemical characteristics of all examined strains of BGC species**.

**Characteristic**	**1**	**2**	**3**	**4**	**6**	**5**	**7**	**8**	**9**	**10**	**11**	**12**	**13**	**14**	**15**	**16**	**17**	**18**	**19**	**20**	**21**	**22**	**23**	**24**	**25**
**GROWTH AT**																									
15°C	+	+	w	+	+	+	+	+	+	−	+	w	+	+	+	+ +	+ +	+ +	+	+	+	+ +	+	+	+
20°C	+	+	ND	+	+	+	+	+	+	ND	ND	+	+	+	+	+ +	+ +	+ +	+	+	+	+ +	+	+	+
37°C	+	−	+	+	w	−	w	−	−	+	+	−	w	w	+	−−	−−	−−	+	w	w	+ −	w	w	W
pH 5	−	−	−	+	−	−	−	−	−	−	−	−	−	−	−	−−	−−	−−	−	−	−	−−	−	−	−
pH 6	+	+	+	+	+	+	+	+	+	+	+	+	+	w	−	−−	+ +	−+	W	w	w	+ +	w	w	−
pH 7	+	+	+	+	+	+	+	+	+	+	+	+	+	−	+	w +	w +	−−	w	w	w	+ −	+	w	+
pH 8	−	w	+	+	−	+	−	−	+	w	−	+	−	−	−	−+	−+	−−	−	−	−	−−	−	−	−
**HYDROLYSIS OF**																									
Tween 60	+	−	+	ND	+	-	+	NG	NG	+	+	+	+	+	+	+ +	+ +	+ +	+	−	−	+ +	+	+	+
Tween 80	−	−	+	+	−	−	−	−	−	+	+	+	−	−	−	−−	−−	−−	−	−	−	−−	−	−	−
**API 20NE**																									
Nitrate reduction	−	+	+	+	−	−	+	−	+	w	+	−	+	+	−	+ +	+ +	−−	−	−	+	−−	−	+	−
Urease	−	−	+	+	−	−	−	−	−	−	−	−	−	−	−	−−	−−	−−	−	−	−	−−	−	−	−
β-Galactosidase	−	+	−	−	−	−	−	−	w	−	−	+	w	−	−	−−	w +	−−	−	−	−	−−	−	−	−
**ASSIMILATION OF**																									
Arabinose	W	+	+	+	w	−	+	−	+	+	w	−	+	+	−	+ w	+ +	+ +	+	+	+	w +	+	+	w
Mannose	+	+	+	+	−	+	+	+	+	+	+	+	+	+	+	+ +	+ +	+ +	+	+	+	+ +	+	+	+
Mannitol	w	+	−	+	w	+	+	+	−	+	+	+	+	+	+	+ +	+ +	+ +	+	+	+	+ +	+	+	+
N-Acetylglucosamine	+	+	+	+	w	+	+	+	+	+	+	+	+	+	+	+ +	+ +	+ +	+	+	+	+ +	+	+	+
Gluconate	+	+	+	+	w	+	+	+	+	+	+	+	+	+	+	+ +	+ +	+ +	+	+	+	+ +	+	+	+
Caprate	+	−	+	−	−	w	+	w	−	−	−	−	−	−	−	−−	− w	w w	−	−	+	−−	+	−	−
Malate	+	W	+	+	w	+	+	+	+	+	+	+	+	+	+	+ w	+ w	+ +	+	+	+	+ +	+	+	+
Citrate	+	−	−	−	−	+	+	+	+	−	w	−	w	−	−	+ w	−−	+ +	−	w	w	w −	−	−	−
Phenylacetate	+	−	+	−	−	+	+	+	−	+	+	+	+	+	+	+ +	+ +	w +	+	+	+	w w	+	+	+
**ENZYME ACTIVITY (API ZYM)**																									
C_4_ lipase	+	+	−	+	w	+	w	+	+	−	+	−	−	+	+	w −	−+	+ +	−	+	−	+ +	w	−	w
C_8_ lipase	w	+	−	+	+	+	w	w	+	−	w	−	−	w	−	−−	− w	− w	−	−	w	w w	−	−	w
Valine arylamidase	w	−	−	−	w	−	−	w	−	−	−	−	−	−	−	−−	−+	− w	−	−	−	+ +	−	−	−
Cystine arylamidase	−	−	−	−	+	−	−	−	−	−	−	−	−	−	−	−−	− w	−−	−	−	−	− w	−	−	−
β-Galactosidase	−	+	−	−	−	−	−	−	+	−	−	−	−	−	−	−−	− w	−−	−	−	−	−−	−	−	−

*Species: 1, B. glathei LMG 14190^T^; 2, B. sordidicola LMG 22029^T^; 3, B. zhejiangensis LMG 27258^T^; 4, B. grimmiae R27^T^; 5, B. choica LMG 22940^T^; 6, B. humi LMG 22934^T^; 7, B. telluris LMG 22936^T^; 8, B. terrestris LMG 22937^T^; 9, B. udeis LMG 27134^T^; 10, B. cordobensis LMG 27620^T^; 11, B. jiangsuensis LMG 27927^T^; 12, B. megalochromosomata LMG 29263^T^; 13, Burkholderia arvi sp. nov. LMG 29317^T^; 14, Burkholderia hypogeia sp. nov. LMG 29322^T^; 15, Burkholderia ptereochthonis sp. nov. LMG 29326^T^; 16, Burkholderia glebae sp. nov. LMG 29325^T^ and LMG 22938; 17, Burkholderia pedi sp. nov. LMG 29323^T^ and R-52605; 18, Burkholderia arationis sp. nov. LMG 29324^T^ and R-23361; 19, Burkholderia fortuita sp. nov. LMG 29320^T^; 20, Burkholderia temeraria sp. nov. LMG 29319^T^; 21, Burkholderia calidae sp. nov. LMG 29321^T^; 22, Burkholderia concitans sp. nov. LMG 29315^T^ and R-46586; 23, Burkholderia turbans sp. nov. LMG 29316^T^; 24, Burkholderia catudaia sp. nov. LMG 29318^T^; 25, Burkholderia peredens sp. nov. LMG 29314^T^. Data for B. glathei, B. sordidicola, B. choica, B. humi, B. telluris, B. terrestris and B. udeis were extracted from Vandamme et al. (2013). Data for B. grimmiae were extracted from Tian et al. (2013). Data for B. cordobensis and B. zhejiangensis were extracted from Draghi et al. (2014). All other data are from the present study. Test results of the type strains are given first, followed by the remaining strains in the order given above. +, present; −, absent; w, weak reaction; v, variable; ND, not determined; NG, no growth*.

## Discussion

While soil is a well-known source of free-living *Burkholderia* species, these organisms often live in close interaction with plants, animals, fungi, or amoebae (Marolda et al., 1999; Van Borm et al., 2002; Kikuchi et al., 2011; Verstraete et al., 2013; Stopnisek et al., 2016; Xu et al., 2016). The BGC represents a poorly known line of descent within the genus *Burkholderia* and most of the 12 validly named BGC species have been isolated from soil. Yet, publicly available sequence data indicate that the taxonomic diversity in this clade is severely underestimated (Nogales et al., 2001; Salles et al., 2006; Pumphrey and Madsen, 2008; Draghi et al., 2014; Verstraete et al., 2014; Peeters et al., 2016; Xu et al., 2016). In the present study, *gyrB* gene sequence analysis was used to screen our strain collection and 17 isolates from human and environmental samples were identified as *B. glathei*-like bacteria. The *gyrB* sequence similarity levels toward other BGC species suggested that the 17 isolates in this study represented 13 novel species (Figure 1). To further characterize the taxonomic status of these isolates, we analyzed the genome sequence of 13 isolates representative for the 13 *gyrB* sequence clusters and of 7 BGC type strains and compared those to 23 whole-genome sequences of BGC strains that were publicly available. Additionally, we also studied their chemotaxonomic and biochemical properties to comply with the polyphasic taxonomic consensus approach to bacterial systematics (Vandamme et al., 1996).

In this genomics era, state-of-the-art sequencing technologies enable direct access to the information contained in whole-genome sequences and it is no longer adequate to deduce genome relatedness through traditional DNA-DNA hybridization experiments (Vandamme and Peeters, 2014; Whitman, 2015). Genomic taxonomy can be studied through various parameters including average nucleotide identity (ANI), GBDP, Maximal Unique Matches index (MUMi), and core gene identity (CGI) (Konstantinidis and Tiedje, 2005; Goris et al., 2007; Deloger et al., 2009; Vanlaere et al., 2009; Meier-Kolthoff et al., 2013). Although, there is a general consensus that genome sequencing could revolutionize prokaryotic systematics (Sutcliffe et al., 2013; Meier-Kolthoff et al., 2014b; Rossello-Mora and Amann, 2015; Thompson et al., 2015), traditional DDH experiments are still being performed and new genome-based methods are evaluated in terms of their correspondence to the existing classifications which are based on DDH data (Wayne et al., 1987; Stackebrandt et al., 2002). The GGDC implementation of the GBDP method provides a quick and reliable alternative to the wet-lab DDH technique and its dDDH prediction capability (including confidence intervals) produces classifications which correlate better with the traditional DDH values than do any of the ANI implementations (Meier-Kolthoff et al., 2013). Among several advantages, GBDP is independent from genome annotation, is applicable to both nucleotide and amino acid data and is immune against problems caused by incompletely sequenced or low-quality draft genomes. Finally, GBDP provides branch support values for the resulting phylogenetic trees (Meier-Kolthoff et al., 2013, 2014a).

We complemented the results of the GBDP analysis with a whole-genome-based phylogeny based on the sequence analysis of 332 single-copy orthologous genes in all BGC genomes. This extended MLSA approach takes only the coding part of the genomes into account and is therefore not influenced by non-coding sequences or pseudogenes that might have a different evolutionary history than the rest of the genome. It depends however on genome annotation, is unable to cope with problems caused by incompletely sequenced or low-quality draft genomes, and its calculations are more compute-intensive and cannot be carried out incrementally. Although, the GBDP and extended MLSA methods used different algorithms, the conclusions drawn from their phylogenies were consistent thus illustrating the robustness of whole-genome based taxonomic methods (Colston et al., 2014).

The GGDC dDDH values and the application of the 70% dDDH cut-off for species delineation (Supplementary Table 1) demonstrated that the 13 clusters delineated through *gyrB* sequence analysis (Figure 1) represented 13 novel BGC species and thus confirmed that *gyrB* gene sequence analysis is a reliable tool for the identification of *Burkholderia* species (Tayeb et al., 2008; Vandamme et al., 2013). Both phylogenomic analyses identified strain MR1, which was isolated from Florida golf course soil and which was shown to reduce the herbicide methylarsenate, as *B. concitans* sp. nov. Next to strain YI23, which was previously identified as *B. cordobensis* by Draghi et al. (2014), the present study also identified strain RPE67, which was isolated from the gut of a stink bug, as *B. cordobensis*. Finally, both phylogenomic analyses also showed that strain PML1(12), an ectomycorrhizosphere-inhabiting bacterium with mineral-weathering ability (Uroz and Oger, 2015), strain S170, a potential plant growth promoter isolated from coniferous forest soil (Llado et al., 2014), strain RPE64, a bacterial symbiont of the bean bug *Riptortus pedestris* (Shibata et al., 2013) and strain Leaf177, an *Arabidopsis* leaf isolate (Bai et al., 2015) all represent novel BGC species.

*Burkholderia* genomes vary in size from 3.75 Mb (*B. rhizoxinica* HKI 454) to 11.3 Mb (*B. terrae* BS001), are characterized by a high G+C content (60–68%) and consist of multiple replicons (Winsor et al., 2008; Ussery et al., 2009). The DNA G+C content of the 13 novel species was calculated from their genome sequences and was in the range of that reported for other BGC species (60–65 mol%). For 10 of the 12 established BGC species, the G+C content was previously calculated by traditional wet-lab methods and the reported values differed by 0.1–3.3 mol% from the values calculated from their genome sequences (Table 5). As reported by Meier-Kolthoff et al., the G+C content calculations based on genome sequences show a higher precision than calculations based on traditional wet-lab methods because the latter methods do not count nucleotides but estimate the genomic G+C content based on the physical properties of the extracted and/or digested genomic DNA (Meier-Kolthoff et al., 2014b). The difference between literature data (Lim et al., 2003; Lu et al., 2012; Tian et al., 2013) and the genome sequence-based G+C content values of *B. sordidicola* LMG 22029^T^, *B. zhejiangensis* OP-1^T^ and *B. grimmiae* R27^T^ is larger than 1% and we therefore present emended descriptions of these species. The genome sizes of the type strains of the 13 novel species ranged from 6.2 Mb (*B. concitans* sp. nov. LMG 29315^T^) to 9.7 Mb (*B. arvi* sp. nov. LMG 29317^T^) and corresponded with the genome sizes of other free-living BGC species (Table 2). Consistent with reductive genome evolution in obligatory symbionts, the smallest BGC genomes belong to the obligatory leaf endosymbionts (2.4–6.2 Mb; Carlier and Eberl, 2012; Carlier et al., 2015; Pinto-Carbo et al., 2016).

**Table 5:** **G+C content (mol%) of validly named BGC species**.

**Strain**	**Wet-lab calculation**	**Calculation from WGS**
*B. glathei* LMG 14190^T^	64.8 (Zolg and Ottow, 1975)	64.7
*B. sordidicola* LMG 22029^T^	61.3 (Lim et al., 2003)	60.2
*B. zhejiangensis* OP-1^T^	59.4 (Lu et al., 2012)	62.7
*B. grimmiae* R27^T^	64.6 (Tian et al., 2013)	63.0
*B. choica* LMG 22940^T^	63 (Vandamme et al., 2013)	62.7
*B. humi* LMG 22934^T^	63 (Vandamme et al., 2013)	62.8
*B. telluris* LMG 22936^T^	64 (Vandamme et al., 2013)	64.0
*B. terrestris* LMG 22937^T^	62 (Vandamme et al., 2013)	62.6
*B. udeis* LMG 27134^T^	60 (Vandamme et al., 2013)	60.0
*B. cordobensis* LMG 27620^T^	63.6 (Draghi et al., 2014)	63.7
*B. jiangsuensis* MP-1^T^	–	62.6
*B. megalochromosomata* JC2949^T^	–	62.7

Biochemically, these novel species are similar to their nearest neighbors. However, tests particularly useful for distinguishing BGC species are growth at 37°C and at pH 8, hydrolysis of tween 60 and 80, nitrate reduction, assimilation of arabinose, caprate and citrate, beta-galactosidase activity and C4 lipase (Table 4). The most discriminating fatty acids are C_16:0_ 3-OH, C_17:0_ cyclo, C_19:0_ cyclo ω8c and summed features 2 and 3 (Table 3). The overall fatty acid profiles of the novel taxa are similar to those of their nearest neighbors and support their placement in the genus *Burkholderia* (Yabuuchi et al., 1992).

The present study again underscores the multifaceted nature of *Burkholderia* bacteria (Coenye and Vandamme, 2003; Mahenthiralingam et al., 2005) and highlights that also BGC species have evolved a broad range of interactions with different hosts. *B. cordobensis* is a striking example of phenotypic and geographic breadth: it was recovered from agricultural soil in Argentina (strain LMG 27620^T^) (Draghi et al., 2014), from golf course soil in South Korea (strain YI23) (Lim et al., 2012) and from the gut of the bean bug *Riptortus pedestris* in Japan (strain RPE67) (Takeshita et al., 2014). The two latter strains (YI23 and RPE67) have fenitrothion degrading properties. The former two strains (LMG 27620^T^ and YI23) were free-living but the latter (RPE67) is an endosymbiont of stink bugs that is not vertically transmitted but acquired from soil by the nymphal insect (Kikuchi et al., 2007). The insecticide resistance to fenitrothion in the pest insects was shown to be established by the endosymbiotic *Burkholderia* strain in the insect gut (Kikuchi et al., 2012) and was shown to emerge as a consequence of repeated insecticide use (Tago et al., 2015). The *Riptortus pedestris*-*B. cordobensis* association thus appears to be a rather young endosymbiosis and contrasts with the symbiosis observed between plant species of the *Rubiaceae* and *Primulaceae* families and several *Candidatus Burkholderia* species. The *Candidatus* designation is a provisional taxonomic status for organisms that have been characterized but that cannot be cultivated at present (Schleifer, 2009). These obligate leaf endosymbionts are vertically transmitted and represent an obligatory symbiosis which was estimated to originate millions of years ago (Lemaire et al., 2011).

BGC species harbor both beneficial and pathogenic strains. Strains PML1(12) and S170 show biotechnological potential for mineral-weathering and plant growth promotion, respectively, and are exemplary for the metabolic versatility of *Burkholderia* organisms (Llado et al., 2014; Uroz and Oger, 2015). Mineral-weathering bacteria dissolute key nutrients from minerals and thereby increase the bioavailability of chemical nutrients in the environment (Uroz et al., 2009). On the other hand, three strains analyzed in the present study were isolated from human clinical samples, i.e., blood, pleural fluid and lung tissue (Table 1) and were classified as two novel species (*Burkholderia concitans* sp. nov. and *Burkholderia turbans* sp. nov.). They represent, to our knowledge, the first examples of human clinical isolates in the *B. glathei* clade. Strikingly, strain MR1, which was isolated from Florida golf course soil and shown to reduce the herbicide methylarsenate, was also identified as *Burkholderia concitans* sp. nov., and this species thus represents yet another human clinical *Burkholderia* species with interesting biotechnological properties (Coenye et al., 2001; Coenye and Vandamme, 2003; Goris et al., 2004; Mahenthiralingam et al., 2005). This study therefore further underscores that there is no phylogenetic subdivision in the genus *Burkholderia* that distinguishes beneficial from pathogenic strains (Angus et al., 2014; Sawana et al., 2014; Estrada-de los Santos et al., 2016; Dobritsa and Samadpour, 2016).

In summary, the present study provides genotypic, chemotaxonomic and phenotypic data which enable the differentiation of 13 novel species in the genus *Burkholderia* and we propose the names *Burkholderia arvi* sp. nov., *Burkholderia hypogeia* sp. nov., *Burkholderia ptereochthonis* sp. nov., *Burkholderia glebae* sp. nov., *Burkholderia pedi* sp. nov., *Burkholderia arationis* sp. nov., *Burkholderia fortuita* sp. nov., *Burkholderia temeraria* sp. nov., *Burkholderia calidae* sp. nov., *Burkholderia concitans* sp. nov., *Burkholderia turbans* sp. nov., *Burkholderia catudaia* sp. nov. and *Burkholderia peredens* sp. nov., with strains LMG 29317^T^, LMG 29322^T^, LMG 29326^T^, LMG 29325^T^, LMG 29323^T^, LMG 29324^T^, LMG 29320^T^, LMG 29319^T^, LMG 29321^T^, LMG 29315^T^, LMG 29316^T^, LMG 29318^T^, and LMG 29314^T^ as type strains, respectively. By making reference cultures and whole-genome sequences of each of these versatile bacteria publicly available, we aim to contribute to future knowledge about the metabolic versatility and pathogenicity of *Burkholderia* organisms.

### Description of *Burkholderia arvi* sp. nov.

*Burkholderia arvi* (ar'vi. L. gen. n. *arvi* of a field).

Cells are Gram-negative, non-motile rods (less than 1 μm wide and about 1 μm long) with rounded ends that occur as single units or in pairs. After 48 h of incubation on trypticase soy agar at 28°C, colonies are round (typically less than 1 mm in diameter), smooth, shiny, non-translucent, with entire margins and a white-creamy color. Grows on MacConkey agar. Growth occurs at 15–37°C and at pH 6–7 in NB at 28°C. Catalase and oxidase activities are present. Hydrolyses tween 60, but not tween 80, starch and casein. When tested using API 20NE strips, positive for nitrate reduction, beta-galactosidase (PNPG) (weak) and assimilation of glucose, arabinose, mannose, mannitol, N-acetyl-glucosamine, gluconate, malate, citrate (weak), and phenylacetate; negative for production of indol, fermentation of glucose, arginine dihydrolase, urease, esculin hydrolysis, gelatin liquefaction and assimilation of maltose, caprate, and adipate. When tested using API ZYM strips, positive for alkaline phosphatase, leucyl arylamidase, acid phosphatase, and phosphoamidase (weak); negative for C4 lipase, C8 lipase, C14 lipase, valine arylamidase, cystine arylamidase, trypsin, chymotrypsin, alpha-galactosidase, beta-galactosidase, beta-glucuronidase, alpha-glucosidase, beta-glucosidase, N-acetyl-beta-glucosaminidase, alpha-mannosidase, and alpha-fucosidase. The following fatty acids are present: C_16:0_, C_16:0_ 3-OH, C_18:1_ ω7c, summed feature 2 (most likely C_14:0_ 3-OH), and summed feature 3 (most likely C_16:1_ ω7c) in moderate amounts (>5%), and C_14:0_, C_16:0_ 2-OH, C_17:0_ cyclo, and C_19:0_ cyclo ω8c in minor amounts (1–5%).

The type strain is LMG 29317^T^ (=CCUG 68412^T^) and was isolated from agricultural soil in Argentina in 2010 (Draghi et al., 2014). Its G+C content is 62.4 mol% (calculated based on its genome sequence). The 16S rRNA, *gyrB* and whole-genome sequence of LMG 29317^T^ are publicly available through the accession numbers LT158615, LT158628, and FCOM02000000, respectively.

### Description of *Burkholderia hypogeia* sp. nov.

*Burkholderia hypogeia* (hy.po.ge'ia. Gr. adj. *hypogeios* subterraneous; N. L. fem. adj. *hypogeia*, subterraneous, earth-born).

Cells are Gram-negative, non-motile rods (about 1 μm wide and 1–2 μm long) with rounded ends that occur as single units or in pairs. After 48 h of incubation on trypticase soy agar at 28°C, colonies are round (typically less than 1 mm in diameter), smooth, shiny, non-translucent, with entire margins and a white-creamy color. Grows on MacConkey agar. Growth occurs at 15–37°C and at pH 6 in NB at 28°C. Catalase and oxidase activities are present. Hydrolyses tween 60, but not tween 80, starch and casein. When tested using API 20NE strips, positive for nitrate reduction and assimilation of glucose, arabinose, mannose, mannitol, N-acetyl-glucosamine, gluconate, malate, and phenylacetate; negative for production of indol, fermentation of glucose, arginine dihydrolase, urease, esculin hydrolysis, gelatin liquefaction, beta-galactosidase (PNPG) and assimilation of maltose, caprate, adipate and citrate. When tested using API ZYM strips, positive for alkaline phosphatase (weak), C4 lipase, C8 lipase (weak), leucyl arylamidase, acid phosphatase and phosphoamidase (weak); negative for C14 lipase, valine arylamidase, cystine arylamidase, trypsin, chymotrypsin, alpha-galactosidase, beta-galactosidase, beta-glucuronidase, alpha-glucosidase, beta-glucosidase, N-acetyl-beta-glucosaminidase, alpha-mannosidase, and alpha-fucosidase. The following fatty acids are present: C_16:0_, C_16:0_ 3-OH, C_17:0_ cyclo, C_18:1_ω7c, summed feature 2 (most likely C_14:0_ 3-OH) and summed feature 3 (most likely C_16:1_ ω7c) in moderate amounts (>5%), and C_14:0_, C_16:0_ 2-OH and C_19:0_ cyclo ω8c in minor amounts (1–5%).

The type strain is LMG 29322^T^ (=CCUG 68407^T^) and was isolated from greenhouse soil in Belgium in 2014 (Peeters et al., 2016). Its G+C content is 63.2 mol% (calculated based on its genome sequence). The 16S rRNA, *gyrB* and whole-genome sequence of LMG 29322^T^ are publicly available through the accession numbers LT158620, LT158633, and FCOA02000000, respectively.

### Description of *Burkholderia ptereochthonis* sp. nov.

*Burkholderia ptereochthonis* (pte.re.o.chtho'nis Gr. n. *pteris* fern; Gr. n. *chthon* soil; N. L. gen. n. *ptereochthonis*, from fern soil).

Cells are Gram-negative, non-motile rods (less than 1 μm wide and about 1 μm long) with rounded ends that occur as single units or in pairs. After 48 h of incubation on trypticase soy agar at 28°C, colonies are round (typically less than 1 mm in diameter), smooth, shiny, non-translucent, with entire margins and a white-creamy color. Grows on MacConkey agar. Growth occurs at 15–37°C and at pH 7 in NB at 28°C. Catalase and oxidase activities are present. Hydrolyses tween 60, but not tween 80, starch and casein. When tested using API 20NE strips, positive for the assimilation of glucose, mannose, mannitol, N-acetyl-glucosamine, gluconate, malate, and phenylacetate; negative for nitrate reduction, production of indol, fermentation of glucose, arginine dihydrolase, urease, esculin hydrolysis, gelatin liquefaction, beta-galactosidase (PNPG) and assimilation of arabinose, maltose, caprate, adipate and citrate. When tested using API ZYM strips, positive for alkaline phosphatase, C4 lipase, leucyl arylamidase, acid phosphatase and phosphoamidase (weak); negative for C8 lipase, C14 lipase, valine arylamidase, cystine arylamidase, trypsin, chymotrypsin, alpha-galactosidase, beta-galactosidase, beta-glucuronidase, alpha-glucosidase, beta-glucosidase, N-acetyl-beta-glucosaminidase, alpha-mannosidase, and alpha-fucosidase. The following fatty acids are present: C_16:0_, C_16:0_ 3-OH, C_17:0_ cyclo, C_18:1_ω7c, summed feature 2 (most likely C_14:0_ 3-OH) and summed feature 3 (most likely C_16:1_ ω7c) in moderate amounts (>5%), and C_14:0_, C_16:0_ 2-OH, C_16:1_ 2-OH, and C_19:0_ cyclo ω8c in minor amounts (1–5%).

The type strain is LMG 29326^T^ (=CCUG 68403^T^) and was isolated from botanical garden soil in Belgium in 2014 (Peeters et al., 2016). Its G+C content is 64.2 mol% (calculated based on its genome sequence). The 16S rRNA, *gyrB* and whole-genome sequence of LMG 29326^T^ are publicly available through the accession numbers LT158624, LT158637, and FCOB02000000, respectively.

### Description of *Burkholderia glebae* sp. nov.

*Burkholderia glebae* (gle'bae. L. gen. n. *glebae* from a lump or clod of earth, soil).

Cells are Gram-negative, non-motile rods (less than 1 μm wide and about 1 μm long) with rounded ends that occur as single units or in pairs. After 48 h of incubation on trypticase soy agar at 28°C, colonies are round, tiny (typically less than 0.5 mm in diameter), non-translucent, with a white-creamy color. Grows on MacConkey agar. Growth occurs at 15–28°C and at pH 7–8 in NB at 28°C (for the type strain only at pH 7). Catalase and oxidase activities are present. Hydrolyses tween 60, but not tween 80, starch and casein. When tested using API 20NE strips, positive for nitrate reduction and assimilation of glucose, arabinose, mannose, mannitol, N-acetyl-glucosamine, gluconate, malate, citrate, and phenylacetate; negative for production of indol, fermentation of glucose, arginine dihydrolase, urease, esculin hydrolysis, gelatin liquefaction, beta-galactosidase (PNPG) and assimilation of maltose, caprate, and adipate. When tested using API ZYM strips, positive for leucyl arylamidase, acid phosphatase and phosphoamidase; negative for C8 lipase, C14 lipase, valine arylamidase, cystine arylamidase, trypsin, chymotrypsin, alpha-galactosidase, beta-galactosidase, beta-glucuronidase, alpha-glucosidase, beta-glucosidase, N-acetyl-beta-glucosaminidase, alpha-mannosidase, and alpha-fucosidase; strain-dependent reactions for alkaline phosphatase (type strain negative) and C4 lipase (type strain weak). The following fatty acids are present in all isolates: C_16:0_, C_16:0_ 3-OH, C_17:0_ cyclo, C_18:1_ω7c, summed feature 2 (most likely C_14:0_ 3-OH), and summed feature 3 (most likely C_16:1_ω7c) in moderate amounts (>5%), and C_14:0_, C_16:0_ 2-OH, C_16:1_ 2-OH, and C_19:0_ cyclo ω8c in minor amounts (1–5%) (mean value of all isolates).

The type strain is LMG 29325^T^ (=CCUG 68404^T^) and was isolated from botanical garden soil in Belgium in 2014 (Peeters et al., 2016). Its G+C content is 62.7 mol% (calculated based on its genome sequence). The 16S rRNA, *gyrB* and whole-genome sequence of LMG 29325^T^ are publicly available through the accession numbers LT158623, LT158636, and FCOJ02000000, respectively. An additional strain has been isolated from soil in the Netherlands (Table 1).

### Description of *Burkholderia pedi* sp. nov.

*Burkholderia pedi* (pe'di. Gr. n. *pedon* soil, earth; N. L. gen. n. *pedi*, from soil).

Cells are Gram-negative, non-motile rods (less than 1 μm wide and 1–2 μm long) with rounded ends that occur as single units or in pairs. After 48 h of incubation on trypticase soy agar at 28°C, colonies are round (typically less than 1 mm in diameter), smooth, shiny, non-translucent, with entire margins and a beige color. Grows on MacConkey agar. Growth occurs at 15–28°C and at pH 6–8 in NB at 28°C (type strain only in pH 6–7). Catalase and oxidase activities are present. Hydrolyses tween 60, but not tween 80, starch and casein. When tested using API 20NE strips, positive for nitrate reduction, beta-galactosidase (PNPG) and assimilation of glucose, arabinose, mannose, mannitol, N-acetyl-glucosamine, gluconate, adipate, malate, and phenylacetate; negative for production of indol, fermentation of glucose, urease, esculin hydrolysis, gelatin liquefaction and assimilation of maltose and citrate; strain-dependent reactions for arginine dihydrolase (type strain negative) and the assimilation of caprate (type strain negative). When tested using API ZYM strips, positive for alkaline phosphatase, leucyl arylamidase, acid phosphatase, and phosphoamidase; negative for C14 lipase, trypsin, chymotrypsin, alpha-galactosidase, beta-glucuronidase, alpha-glucosidase, beta-glucosidase, alpha-mannosidase, and alpha-fucosidase; strain-dependent reactions for C4 lipase (type strain negative), C8 lipase (type strain negative), valine arylamidase (type strain negative), cystine arylamidase (type strain negative), beta-galactosidase (type strain negative), and N-acetyl-beta-glucosaminidase (type strain negative). The following fatty acids are present in all isolates: C_16:0_, C_16:0_ 3-OH, C_17:0_ cyclo, C_18:1_ω7c, summed feature 2 (most likely C_14:0_ 3-OH), and summed feature 3 (most likely C_16:1_ω7c) in moderate amounts (>5%), and C_14:0_, C_16:0_ 2-OH, C_16:1_ 2-OH, and C_19:0_ cyclo ω8c in minor amounts (1–5%) (mean value of all isolates).

The type strain is LMG 29323^T^ (=CCUG 68406^T^) and was isolated from greenhouse soil in Belgium in 2014 (Peeters et al., 2016). Its G+C content is 63.0 mol% (calculated based on its genome sequence). The 16S rRNA, *gyrB*, and whole-genome sequence of LMG 29323^T^ are publicly available through the accession numbers LT158621, LT158634, and FCOE02000000, respectively. An additional strain has been isolated from the same sample (Table 1).

### Description of *Burkholderia arationis* sp. nov.

*Burkholderia arationis* (a.ra.ti.o'nis. L. gen. n. *arationis* from a field).

Cells are Gram-negative, non-motile rods (less than 1 μm wide and about 1 μm long) with rounded ends that occur as single units or in pairs. After 48 h of incubation on trypticase soy agar at 28°C, colonies are round (typically less than 1 mm in diameter), smooth, shiny, translucent, with entire margins and a white-creamy color. Grows on MacConkey agar. Growth occurs at 15–28°C and at pH 6 in NB at 28°C (the type strain did not grow in liquid NB medium). Catalase and oxidase activities are present. Hydrolyses tween 60, but not tween 80, starch and casein. When tested using API 20NE strips, positive for assimilation of glucose, arabinose, mannose, mannitol, N-acetyl-glucosamine, gluconate, caprate (weak), adipate (weak), malate, citrate, and phenylacetate; negative for nitrate reduction, production of indol, fermentation of glucose, arginine dihydrolase, urease, esculin hydrolysis, gelatin liquefaction, beta-galactosidase (PNPG), and assimilation of maltose. When tested using API ZYM strips, positive for alkaline phosphatase, C4 lipase, leucyl arylamidase, acid phosphatase and phosphoamidase; negative for C14 lipase, cystine arylamidase, trypsin, alpha-galactosidase, beta-galactosidase, beta-glucuronidase, alpha-glucosidase, beta-glucosidase, N-acetyl-beta-glucosaminidase, alpha-mannosidase, and alpha-fucosidase; strain-dependent reactions for C8 lipase (type strain negative), valine arylamidase (type strain negative), and chymotrypsin (type strain negative). The following fatty acids are present in all isolates: C_16:0_, C_16:0_ 3-OH, C_18:1_ω7c, summed feature 2 (most likely C_14:0_ 3-OH), and summed feature 3 (most likely C_16:1_ ω7c) in moderate amounts (>5%), and C_14:0_ in minor amounts (1-5%) (mean value of all isolates).

The type strain is LMG 29324^T^ (=CCUG 68405^T^) and was isolated from botanical garden soil in Belgium in 2014 (Peeters et al., 2016). Its G+C content is 62.8 mol% (calculated based on its genome sequence). The 16S rRNA, *gyrB*, and whole-genome sequence of LMG 29324^T^ are publicly available through the accession numbers LT158622, LT158635, and FCOG02000000, respectively. An additional strain has been isolated from soil in the Netherlands (Table 1).

### Description of *Burkholderia fortuita* sp. nov.

*Burkholderia fortuita* (for.tu.i'ta. L. fem. adj. *fortuita* accidental, unpremeditated; referring to its fortuitous isolation when searching for *Burkholderia caledonica* endophytes).

Cells are Gram-negative, non-motile rods (less than 1 μm wide and about 1 μm long) with rounded ends that occur as single units or in pairs. After 48 h of incubation on trypticase soy agar at 28°C, colonies are round (typically less than 1 mm in diameter), smooth, shiny, non-translucent, with entire margins and a beige color. Grows on MacConkey agar. Growth occurs at 15–37°C and at pH 6–7 in NB at 28°C. Catalase and oxidase activities are present. Hydrolyses tween 60, but not tween 80, starch and casein. When tested using API 20NE strips, positive for the assimilation of glucose, arabinose, mannose, mannitol, N-acetyl-glucosamine, gluconate, malate, and phenylacetate; negative for nitrate reduction, production of indol, fermentation of glucose, arginine dihydrolase, urease, esculin hydrolysis, gelatin liquefaction, beta-galactosidase (PNPG) and assimilation of maltose, caprate, adipate, and citrate. When tested using API ZYM strips, positive for alkaline phosphatase (weak), leucyl arylamidase, acid phosphatase, and phosphoamidase (weak); negative for C4 lipase, C8 lipase, C14 lipase, valine arylamidase, cystine arylamidase, trypsin, chymotrypsin, alpha-galactosidase, beta-galactosidase, beta-glucuronidase, alpha-glucosidase, beta-glucosidase, N-acetyl-beta-glucosaminidase, alpha-mannosidase, and alpha-fucosidase. The following fatty acids are present: C_16:0_, C_16:0_ 3-OH, C_17:0_ cyclo, C_18:1_ω7c, summed feature 2 (most likely C_14:0_ 3-OH), and summed feature 3 (most likely C_16:1_ ω7c) in moderate amounts (>5%), and C_14:0_, C_16:0_ 2-OH, C_16:1_ 2-OH, and C_19:0_ cyclo ω8c in minor amounts (1–5%).

The type strain is LMG 29320^T^ (=CCUG 68409^T^) and was isolated from *Fadogia homblei* rhizosphere soil in South Africa in 2013 (Verstraete et al., 2014). Its G+C content is 62.9 mol% (calculated based on its genome sequence). The 16S rRNA, *gyrB* and whole-genome sequence of LMG 29320^T^ are publicly available through the accession numbers LT158618, LT158631, and FCNX02000000, respectively.

### Description of *Burkholderia temeraria* sp. nov.

*Burkholderia temeraria* (te.me.ra'ri.a. L. fem. adj. *temeraria* accidental, inconsiderate; referring to its accidental isolation when searching for *Burkholderia caledonica* endophytes).

Cells are Gram-negative, non-motile rods (less than 1 μm wide and about 1 μm long) with rounded ends that occur as single units or in pairs. After 48 h of incubation on trypticase soy agar at 28°C, colonies are round (typically less than 1 mm in diameter), smooth, shiny, non-translucent, with entire margins and a white-creamy color. Grows on MacConkey agar. Growth occurs at 15–37°C and at pH 6–7 in NB at 28°C. Catalase and oxidase activities are present. Does not hydrolyze tween 60, tween 80, starch and casein. When tested using API 20NE strips, positive for the assimilation of glucose, arabinose, mannose, mannitol, N-acetyl-glucosamine, gluconate, malate, citrate (weak), and phenylacetate; negative for nitrate reduction, production of indol, fermentation of glucose, arginine dihydrolase, urease, esculin hydrolysis, gelatin liquefaction, beta-galactosidase (PNPG) and assimilation of maltose, caprate, and adipate. When tested using API ZYM strips, positive for alkaline phosphatase, C4 lipase, leucyl arylamidase, acid phosphatase, and phosphoamidase (weak); negative for C8 lipase, C14 lipase, valine arylamidase, cystine arylamidase, trypsin, chymotrypsin, alpha-galactosidase, beta-galactosidase, beta-glucuronidase, alpha-glucosidase, beta-glucosidase, N-acetyl-beta-glucosaminidase, alpha-mannosidase, and alpha-fucosidase. The following fatty acids are present: C_16:0_, C_16:0_ 3-OH, C_17:0_ cyclo, C_18:1_ω7c, summed feature 2 (most likely C_14:0_ 3-OH) and summed feature 3 (most likely C_16:1_ ω7c) in moderate amounts (>5%), and C_14:0_, C_16:0_ 2-OH, and C_19:0_ cyclo ω8c in minor amounts (1–5%).

The type strain is LMG 29319^T^ (=CCUG 68410^T^) and was isolated from *Fadogia homblei* rhizosphere soil in South Africa in 2013 (Verstraete et al., 2014). Its G+C content is 62.7 mol% (calculated based on its genome sequence). The 16S rRNA, *gyrB* and whole-genome sequence of LMG 29319^T^ are publicly available through the accession numbers LT158617, LT158630, and FCOI02000000, respectively.

### Description of *Burkholderia calidae* sp. nov.

*Burkholderia calidae* (ca'li.dae. L. gen. n. *calidae* from warm water, because this strain was isolated from pond water in a tropical garden).

Cells are Gram-negative, non-motile rods (about 1 μm wide and 1 μm long) with rounded ends that occur as single units or in pairs. After 48 h of incubation on trypticase soy agar at 28°C, colonies are round (typically about 1 mm in diameter), smooth, shiny, non-translucent, with entire margins and a white-creamy color. Grows on MacConkey agar. Growth occurs at 15–37°C and at pH 6–7 in NB at 28°C. Catalase and oxidase activities are present. Does not hydrolyze tween 60, tween 80, starch and casein. When tested using API 20NE strips, positive for nitrate reduction and assimilation of glucose, arabinose, mannose, mannitol, N-acetyl-glucosamine, gluconate, caprate, malate, citrate (weak), and phenylacetate; negative for production of indol, fermentation of glucose, arginine dihydrolase, urease, esculin hydrolysis, gelatin liquefaction, beta-galactosidase (PNPG) and assimilation of maltose and adipate. When tested using API ZYM strips, positive for alkaline phosphatase (weak), C8 lipase (weak), leucyl arylamidase (weak), acid phosphatase and phosphoamidase (weak); negative for C4 lipase, C14 lipase, valine arylamidase, cystine arylamidase, trypsin, chymotrypsin, alpha-galactosidase, beta-galactosidase, beta-glucuronidase, alpha-glucosidase, beta-glucosidase, N-acetyl-beta-glucosaminidase, alpha-mannosidase, and alpha-fucosidase. The following fatty acids are present: C_16:0_, C_18:1_ω7c, summed feature 2 (most likely C_14:0_ 3-OH) and summed feature 3 (most likely C_16:1_ ω7c) in moderate amounts (>5%), and C_14:0_, C_16:0_ 2-OH, C_16:0_ 3-OH, and C_17:0_ cyclo in minor amounts (1–5%).

The type strain is LMG 29321^T^ (=CCUG 68408^T^) and was isolated from greenhouse pond water in Belgium in 2013 (Peeters et al., 2016). Its G+C content is 62.5 mol% (calculated based on its genome sequence). The 16S rRNA, *gyrB* and whole-genome sequence of LMG 29321^T^ are publicly available through the accession numbers LT158619, LT158632, and FCOX02000000, respectively.

### Description of *Burkholderia concitans* sp. nov.

*Burkholderia concitans* (con.ci'tans. L. fem. part. pres. *concitans* disturbing, upsetting; because the isolation of this bacterium from human sources, including blood, further disturbs the image of this lineage of *Burkholderia* species as benign bacteria).

Cells are Gram-negative, non-motile rods (less than 1 μm wide and about 1 μm long) with rounded ends that occur as single units or in pairs. After 48 h of incubation on trypticase soy agar at 28°C, colonies are round (typically less than 1 mm in diameter), smooth, shiny, non-translucent, with entire margins and a white-creamy color. Grows on MacConkey agar. Growth occurs at 15–28°C (additionally, the type strains grows at 37°C) and at pH 6–7 in NB at 28°C. Catalase and oxidase activities are present. Hydrolyses tween 60, but not tween 80, starch and casein. When tested using API 20NE strips, positive for the assimilation of glucose, arabinose, mannose, mannitol, N-acetyl-glucosamine, gluconate, malate, and phenylacetate; negative for nitrate reduction, production of indol, fermentation of glucose, arginine dihydrolase, urease, esculin hydrolysis, gelatin liquefaction, beta-galactosidase (PNPG) and assimilation of maltose, caprate, and adipate; strain-dependent reactions for the assimilation of citrate (type strain weak). When tested using API ZYM strips, positive for alkaline phosphatase, C4 lipase, C8 lipase (weak), leucyl arylamidase, valine arylamidase, acid phosphatase, and phosphoamidase; negative for C14 lipase, trypsin, chymotrypsin, alpha-galactosidase, beta-galactosidase, beta-glucuronidase, alpha-glucosidase, beta-glucosidase, N-acetyl-beta-glucosaminidase, alpha-mannosidase, and alpha-fucosidase; strain-dependent reactions for cystine arylamidase (type strain negative). The following fatty acids are present in all isolates: C_16:0_, C_16:0_ 3-OH, C_17:0_ cyclo, C_18:1_ω7c, C_19:0_ cyclo ω8c, summed feature 2 (most likely C_14:0_ 3-OH) and summed feature 3 (most likely C_16:1_ ω7c) in moderate amounts (>5%), and C_14:0_, C_16:0_ 2-OH, and C_16:1_ 2-OH in minor amounts (1–5%) (mean value of all isolates).

The type strain is LMG 29315^T^ (=CCUG 68414^T^) and was isolated from human lung tissue in the USA in 2006. Its G+C content is 63.2 mol%. The 16S rRNA, *gyrB*, and whole-genome sequence of LMG 29315^T^ are publicly available through the accession numbers LT158613, LT158626 and FCNV02000000, respectively. An additional strain has been isolated from human blood in the USA in 2010 (Table 1).

### Description of *Burkholderia turbans* sp. nov.

*Burkholderia turbans* (tur'bans. L. fem. part. pres. *turbans* disturbing, agitating, because the isolation of this bacterium from human pleural fluid further disturbs the image of this lineage of *Burkholderia* species as benign bacteria).

Cells are Gram-negative, non-motile rods (about 1 μm wide and 1–1.5 μm long) with rounded ends that occur as single units or in pairs. After 48 h of incubation on trypticase soy agar at 28°C, colonies are round (typically less than 1 mm in diameter), smooth, shiny, non-translucent, with entire margins and a white-creamy color. Grows on MacConkey agar. Growth occurs at 15–37°C and at pH 6–7 in NB at 28°C. Catalase and oxidase activities are present. Hydrolyses tween 60, but not tween 80, starch and casein. When tested using API 20NE strips, positive for the assimilation of glucose, arabinose, mannose, mannitol, N-acetyl-glucosamine, gluconate, caprate, malate, and phenylacetate; negative for nitrate reduction, production of indol, fermentation of glucose, arginine dihydrolase, urease, esculin hydrolysis, gelatin liquefaction, beta-galactosidase (PNPG) and assimilation of maltose, adipate and citrate. When tested using API ZYM strips, positive for alkaline phosphatase, C4 lipase (weak), leucyl arylamidase, acid phosphatase, and phosphoamidase (weak); negative for C8 lipase, C14 lipase, valine arylamidase, cystine arylamidase, trypsin, chymotrypsin, alpha-galactosidase, beta-galactosidase, beta-glucuronidase, alpha-glucosidase, beta-glucosidase, N-acetyl-beta-glucosaminidase, alpha-mannosidase, and alpha-fucosidase. The following fatty acids are present: C_16:0_, C_17:0_ cyclo, C_18:1_ω7c, summed feature 2 (most likely C_14:0_ 3-OH) and summed feature 3 (most likely C_16:1_ ω7c) in moderate amounts (>5%), and C_14:0_, C_16:0_ 2-OH, C_16:0_ 3-OH, C_16:1_ 2-OH, and C_19:0_ cyclo ω8c in minor amounts (1–5%).

The type strain is LMG 29316^T^ (=CCUG 68413^T^) and was isolated from human pleural fluid in the USA in 2006. Its G+C content is 63.1 mol% (calculated based on its genome sequence). The 16S rRNA, *gyrB* and whole-genome sequence of LMG 29316^T^ are publicly available through the accession numbers LT158614, LT158627, and FCOD02000000, respectively.

### Description of *Burkholderia catudaia* sp. nov.

*Burkholderia catudaia* (ca.tu.da'ia. Gr. adj. *catudaios* subterraneous; N. L. fem. adj. *catudaia*, earth-born).

Cells are Gram-negative, non-motile rods (about 1 μm wide and 1–2 μm long) with rounded ends that occur as single units or in pairs. After 48 h of incubation on trypticase soy agar at 28°C, colonies are round (typically less than 1 mm in diameter), smooth, shiny, non-translucent, with entire margins and a white-creamy color. Grows on MacConkey agar. Growth occurs at 15–37°C and at pH 6–7 in NB at 28°C. Catalase and oxidase activities are present. Hydrolyses tween 60, but not tween 80, starch and casein. When tested using API 20NE strips, positive for nitrate reduction and assimilation of glucose, arabinose, mannose, mannitol, N-acetyl-glucosamine, gluconate, malate, and phenylacetate; negative for production of indol, fermentation of glucose, arginine dihydrolase, urease, esculin hydrolysis, gelatin liquefaction, beta-galactosidase (PNPG) and assimilation of maltose, caprate, adipate, and citrate. When tested using API ZYM strips, positive for alkaline phosphatase (weak), leucyl arylamidase, acid phosphatase, and phosphoamidase (weak); negative for C4 lipase, C8 lipase, C14 lipase, valine arylamidase, cystine arylamidase, trypsin, chymotrypsin, alpha-galactosidase, beta-galactosidase, beta-glucuronidase, alpha-glucosidase, beta-glucosidase, N-acetyl-beta-glucosaminidase, alpha-mannosidase, and alpha-fucosidase. The following fatty acids are present: C_16:0_, C_16:0_ 3-OH, C_18:1_ω7c, summed feature 2 (most likely C_14:0_ 3-OH) and summed feature 3 (most likely C_16:1_ ω7c) in moderate amounts (>5%), and C_14:0_, C_16:0_ 2-OH, C_17:0_ cyclo, and C_19:0_ cyclo ω8c in minor amounts (1–5%).

The type strain is LMG 29318^T^ (=CCUG 68411^T^) and was isolated from *Fadogia homblei* rhizosphere soil in South Africa in 2013 (Verstraete et al., 2014). Its G+C content is 62.8 mol% (calculated based on its genome sequence). The 16S rRNA, *gyrB* and whole-genome sequence of LMG 29318^T^ are publicly available through the accession numbers LT158616, LT158629, and FCOF02000000, respectively.

### Description of *Burkholderia peredens* sp. nov.

Burkholderia *peredens* (per.e'dens. L. fem. part. pres. *peredens* consuming, devouring; referring to the capacity of this bacterium to degrade fenitrothion).

Cells are Gram-negative, non-motile rods (about 1 μm wide and 1–2 μm long) with rounded ends that occur as single units or in pairs. After 48 h of incubation on trypticase soy agar at 28°C, colonies are round (typically less than 1 mm in diameter), smooth, shiny, non-translucent, with entire margins and a white-creamy color. Grows on MacConkey agar. Growth occurs at 15–37°C and at pH 7 in NB at 28°C. Catalase and oxidase activities are present. Hydrolyses tween 60, but not tween 80, starch and casein. When tested using API 20NE strips, positive for the assimilation of glucose, arabinose (weak), mannose, mannitol, N-acetyl-glucosamine, gluconate, malate, and phenylacetate; negative for nitrate reduction, production of indol, fermentation of glucose, arginine dihydrolase, urease, esculin hydrolysis, gelatin liquefaction, beta-galactosidase (PNPG) and assimilation of maltose, caprate, adipate, and citrate. When tested using API ZYM strips, positive for alkaline phosphatase, C4 lipase (weak), C8 lipase (weak), leucyl arylamidase, acid phosphatase, and phosphoamidase (weak); negative for C14 lipase, valine arylamidase, cystine arylamidase, trypsin, chymotrypsin, alpha-galactosidase, beta-galactosidase, beta-glucuronidase, alpha-glucosidase, beta-glucosidase, N-acetyl-beta-glucosaminidase, alpha-mannosidase, and alpha-fucosidase. The following fatty acids are present: C_16:0_, C_16:0_ 3-OH, C_18:1_ω7c, summed feature 2 (most likely C_14:0_ 3-OH) and summed feature 3 (most likely C_16:1_ ω7c) in moderate amounts (>5%), and C_14:0_, C_16:0_ 2-OH, C_16:1_ 2-OH and C_17:0_ cyclo in minor amounts (1–5%).

The type strain is LMG 29314^T^ (=CCUG 68415^T^) and was isolated from soil in Japan (Hayatsu et al., 2000). Its G+C content is 63.1 mol% (calculated based on its genome sequence). The 16S rRNA, *gyrB* and whole-genome sequence of LMG 29314^T^ are publicly available through the accession numbers LT158612, LT158625, and FCOH02000000, respectively.

### Emended description of the species *Burkholderia sordidicola* (Lim et al., 2003)

The description of the species *Burkholderia sordidicola* is the one given by Lim et al. (2003) with the following modification. The G+C content of the type strain is 60.2%.

### Emended description of the species *Burkholderia zhejiangensis* (Lu et al., 2012)

The description of the species *Burkholderia zhejiangensis* is the one given by Lu et al. (2012) with the following modification. The G+C content of the type strain is 62.7%.

### Emended description of the species *Burkholderia grimmiae* (Tian et al., 2013)

The description of the species *Burkholderia grimmiae* is the one given by Tian et al. (2013) with the following modification. The G+C content of the type strain is 63.0%.

## Author contributions

CP carried out the genomic data analysis and drafted the manuscript. JM performed all GBDP-related analyses. BV participated in the ortholog analysis and whole-genome based phylogeny. ED performed the DNA extractions, fatty acid analysis, and biochemical characterization. VC directed the genomic sequencing methods and initial analysis. PV conceived of the study, participated in the design and coordination and helped writing the manuscript. All authors read and approved the final manuscript.

### Conflict of interest statement

The authors declare that the research was conducted in the absence of any commercial or financial relationships that could be construed as a potential conflict of interest.

## Supplementary material

Supplementary Table1 **Pairwaise GGD and dDDH values**. For each pair of genomes the intergenomic distance (GGD), dDDH and 95% confidence interval (CI) is given, sorted from high to low dDDH. dDDH values above the 70% cut-off for species delineation are shown in bold type.

## Abbreviations

BGC: *Burkholderia glathei* clade
GGDC: Genome-to-Genome Distance Calculator
GBDP: Genome Blast Distance Phylogeny
dDDH: digital DNA-DNA hybridization
MLSA: multilocus sequence analysis.
